# Carcinogens in Food: Evaluating the Presence of Cadmium, Lead, in Poultry Meat in South India

**DOI:** 10.31557/APJCP.2021.22.11.3507

**Published:** 2021-11

**Authors:** Manikantan Pappuswamy, Arun Meyyazhagan, Balamuralikrish Balasubramanian, Haripriya Kuchi Bhotla, Karthika Pushparaj, Murugesh Easwaran, Vijaya Anand Arumugam, Thirunavukkarasu Periyaswamy, Karthick Dhandapani

**Affiliations:** 1 *Departmen of Life Sciences, CHRIST (Deemed to be University), Bangalore, Karnataka, India. *; 2 *Department of Food Science and Biotechnology, College of Life Science, Sejong University, Seoul 05006, Republic of Korea. *; 3 *Bioknowl Insights Private Limited, Coimbatore, Tamil Nadu 641046, India. *; 4 *Department of Zoology, School of Bioscience, Avinashilingam Institute for Home Sciences and Higher Education for Women , Coimbatore, Tamil Nadu, India. *; 5 *Computational Biology Laboratory, Department of Bioinformatics, Bharathiar University, Coimbatore-641046, Tamil Nadu, India. *; 6 *Department of Human Genetics and Molecular Biology, Bharathiar University, Coimbatore, Tamil Nadu, India.*; 7 *Nehru Arts & Science College (Affiliated to Bharathiar University), Coimbatore, Tamilnadu, India. *; 8 *School of Engineering, Shanghai Jiao Tong University, Shanghai 200241, China. *

**Keywords:** Poultry chicken, toxic effects, Lead, Cadmium and Zinc

## Abstract

**Objective:**

Local chickens were spontaneously sampled and slaughtered in the central markets of Coimbatore, Erode, and Namakkal districts, South India.

**Materials and Methods:**

Wet digestion was used to extract lead (Pb), cadmium (Cd), and zinc (Zn) in their blood and selected different organs (intestine, breast, liver, and gizzard), and their concentrations were measured using an atomic absorption spectrophotometer.

**Results:**

Apart from the blood of chickens from Coimbatore and Namakkal, where Pb was not found, the concentrations of Pb in the blood and organs of chickens from the three towns ranged from 1.8 to 8.33 mg/kg, exceeding the maximum tolerance thresholds (0.1 mg/kg) in internal organs of poultry birds. Except for the intestine of chickens from the three areas, Cd was only found in the heart, blood, and gizzard of Erode chickens, as well as the liver and gizzard of Namakkal chickens, in concentrations ranging from 0.13 to 0.58. According to threshold level, the upper limit met the maximum limits (0.5 mg/kg). Zn was found in all sections of chickens from the three selected districts, with concentrations ranging from 4.96 to 174.17 mg/kg.

**Conclusion:**

Its concentrations were within the permissible limits (10-50 mg/kg) in some areas of certain chickens, but it surpassed the permissible limit in the liver of chicken from Coimbatore. Any organs and blood from local chickens sold in Coimbatore, Erode, and Namakkal areas can be hazardous to one’s health.

## Introduction

Chicken and chicken related food sources are rich nutritive for human health and development. It is a primary source of various energy molecules, vitamins, minerals, delicious and affordable intake as daily allowances. (Attia et al, 2016). Though, products from poultry industry may cause serious risk factors due to the contamination with hazardous metals through the food cycle and outer environment (Bamuwamye et al, 2015; Chakraborty, 2020). While feeding of these poultry farm mainly enhanced some elements rich food sources such as Fe, Mn, Zn and Cu for better growth in minimal period of time. (Doyle and Spaulding 1978; Martz, 1981).

These products are rich in B and B12 vitamins also it contains the important trace elements especially zinc and iron which is not present in natural plant sources (Eton et al, 2008). It also contains essential antioxidant selenium which has been related to reduce the risk of cardiac related defects and certain type of cancers (Pappas et al, 2006). Moreover, the meat products are containing high concentration of protein and their building blocks amino acid composition which compensates the short fall of food sources (Abduljaleel et al, 2012). Chicken meat farm is predominantly producing and selling in large quantity in Tamilnadu, Southern India, particularly local and central markets (Narasimhan and Gireesh Babu, 2012). It has been major sources of protein to the ever-growing lower economic population making significant development to nutritive factor as well as their economic growth balance (IEGI, 2013). Totally more than millions of tonnes of chicken and 8 million of eggs produced per day in selected demographic area and supply to the nearby districts (The Hindu, 2015; Malarvizhi and Geetha, 2015; Jacob et al, 1998).

According to Khan et al., (2015) metal and metallic elements are natural minimal constituents of the earth’s crust found in the ecosystem. Trace elements are also found in food additives and it provide nutritional as well as toxicological importance in our system. Furthermore, sodium, potassium, and calcium are very vital for induce the metabolic activity for human, whereas lead and cadmium which are effects to the metabolic pathway even in lower concentration. On the other hand, iron, copper and zinc which are trace heavy metals requires low quantity to normal metabolic pathways, but it pose to health risk when at higher level (Eton et al, 2008).

There are four main kinds of possibly carcinogenic chemicals that have been studied in people to see whether they cause cancer. The first are natural compounds that are unavoidably present in food and meat. Second, the IARC Monographs on the Evaluation of Carcinogenic Risks to Humans (http://monographs.iarc.fr/) are published by the International Agency for Research on Cancer (IARC). (Abnet, 2007) “The Monographs constitute the first stage in carcinogenic risk assessment, which includes evaluation of all relevant material in order to evaluate the strength of the existing evidence that particular exposures may change the incidence of cancer in humans, especially heavy metals” according to the IARC. (USNTP, 2002)

Moreover, human cancer risk estimates are mainly based on data from human epidemiologic research or laboratory experiments on experimental animals. Epidemiologic research has the benefit of directly informing people about carcinogenic hazards such as heavy metals (Ames and Gold, 1990).

Typically, these heavy metals enter to the system inclusively industrial wastes, plant irrigation with effluent water bodies and contaminated soils. Recent reports by Ping et al. (2014) found out that potential source of heavy metals in biological system through the contaminated soil. Accumulation of these heavy metals in poultry industry through food sources such as plants grown in contaminated soil and water intake (Caudrado et al., 1995). Poultry bird’s food chain may also alter by exposure of heavy metals through food and quality of environmental contamination in breeding place. This is the major way of heavy metal effects and ingestion to human body. Heavy metal contamination issue is in global concern because it occupies the high risk to human health, safety of food in general. Recent studies shows that heavy metals alter the genetic makeup of DNA and induce the certain changes of the immune system and intensify the susceptibility to disease infection (Mansour, 2014; Jaishankar et al., 2014; Akan et al., 2010; Ogwok et al., 2014). Moreover, it accumulates into biological system and effects the whole system then biomagnifying through metabolic pathway and food chain. According to Cuadrado et al., (1995) reports, meat and meat products are main sources of heavy metal intake. Studies insist that maintain the free range of these meat products and control the exposure level with proper waste disposal system around the poultry farm. In view of the entire issues, present study was focused on analyse the heavy metal concentration in chickens in local markets in three selected districts of Tamilnadu, South India.

## Materials and Methods

*Selected Area*

*Sampling*

Chickens were collected from the local and central markets in Coimbatore, Erode and Namakkal districts in Tamilnadu, Southern India. The chickens are dissected by stainless sharp knife and cut the throat in sterile condition. The 5 ml of blood was collected into an EDTA tube and stored in freezers until used.

*Sample preparation and digestion*

*Solid sample digestion*

Solid organs (heart, gizzard, liver and intestine) were cut into small pieces and dried in hot air oven at 106^o^C for 72 hrs then crushed the sample with mixer. All the crushed specimen was kept in a bag covered by polythene then transferred and stored in a sterilized desiccator. Solubilise the heavy metals by digest wet condition and 1 g dried sample of intestine, gizzard and liver separated for analysis as well as 0.5 g of heart taken and mix with 10 mL of 3:2 65% v/v and 70% v/v HCIO4 in a 100 mL beaker. The sample beakers gently swirled and incubated to 12-14 hrs. Then it was digested for 3 hrs in 70^o^C water bath, every 30 min it allows to be swirling for ensure that proper digestion. Fully allowed to cool and filtered into a 50 mL volumetric flask and volume made up to the mark. Finally, each sample was transferred to sample bottle for heavy metal analysis. 

*Liquid sample digestion*

A 0.5 mL of solution were prepared in the ratio 20:1 (HNO3 and H2SO4) was added 1.5 mL of blood sample and 10mL of water. The mixture was thoroughly mixed vortex and incubate in water bath at 60^o^C until the mixture were reached half of its total volume. Additionally added 1 mL of HNO3 and heated until the fully evaporated and clear. Then cooled in room temperature. Filtered into a proper volumetric flask (50mL) using 120 mm Whatman No.1 filter paper and mark up to the volume. Finally, samples were stored in freezer until analyse for heavy metals. 

*Elemental analysis of samples*

Heavy metals concentration in digestates of each sample were determined using Atomic Absorption Spectrophotometer with standard protocol. For every heavy metal, machine was set up proper cathode lamp at a specific wavelength for the heavy metal analysis. The absorbance was measured at 15 mA of lamp and the peak height mode of the wavelengths used were 283.3 nm, 228.8 nm, 213.9 nm for lead, cadmium and zinc respectively. 

*Statistical Analysis*

Mean and standard deviations were analysed by proper SPSS 20.0 method. Control and experimental samples were compared to heavy metals in each selected sample as well as among all the samples using one-way analysis of variance (ANOVA). Significant values were measured by p=0.05 to analyse the significance of variation.

## Results

The mean concentrations of metals in chicken organs (intestine, heart, liver and gizzard) and blood samples from the three towns (Coimbatore, Erode and Namakkal) are shown in Table 1. Also, Table 2 shows the concentrations of heavy metals in internal tissues and organs of poultry birds from similar studies and this study while the maximum tolerance limits of some heavy metals in the internal tissues and organs of poultry birds are shown. Blood samples were analysed in only from Erode district other than Coimbatore and Namakkal. High level of lead level noted in Coimbatore and Erode than Namakkal due to high level of anthropogenic effects and highly populated in these areas. 

The trends generally observed for lead are Coimbatore > Namakkal > Erode for intestine and heart, Coimbatore>Erode>Namakkal for liver and Erode>Coimbatore>Namakkal for gizzard Table 1. Gizzard of chicken samples from Namakkal also had the lowest lead mean concentration (2.04 mg/kg) while the highest occurred in heart of chicken from Coimbatore (6.42 mg/kg). Lead for this study ranged as 2.40-3.43 mg/kg for intestine, 2.02-6.43 mg/kg for heart, 2.30-5.46 mg/kg for liver, 1.68-5.44 mg/kg for gizzard. The trend was heart>liver>gizzard>intestine>blood (Table 2),

In this study, the respective cadmium concentrations obtained for intestine, heart and blood were 0.67-0.50 mg/kg, 0.12 mg/kg and 0.11 mg/kg (Table 1). Cadmium was highest in gizzard of chicken from Namakkal (0.72 mg/kg) and lowest in blood of chicken from Erode (0.12 mg/kg), the blood samples where it was only detected. Where detected, the trend for cadmium in chicken was Namakkal>Coimbatore>Erode. No significant difference was observed in cadmium concentration for chicken intestine from Erode and Namakkal whereas a significant difference existed in gizzard of chicken from Erode and Namakkal (p < 0.05). Similar studies vary in their reports on cadmium (Table 2). In one of such, cadmium was not detected in the heart while it was 0.28 mg/kg for this study. The range of cadmium concentration in liver for two studies was from 0.007 to 0.23 mg/kg while 0.3 mg/kg was observed for this study. Similarly, cadmium in gizzard ranged from 0.01 to 1.02 mg/kg in a study whereas in this study, the range was 0.29 to 0.56 mg/kg.

**Table 1:** Concentration of Heavy Metals in Chicken Blood and Organs from Coimbatore, Erode and Namakkal

Organs of Chicken	Name of the Districts								
	Coimbatore			Erode			Namakkal		
	Level of Metals (mg/kg)								
	Pb	Cd	Zn	Pb	Cd	Zn	Pb	Cd	Zn
Intestine	5.22±0.64	0.50±0.45	65.00±10.34	4.46±0.62	0.34±0.39	47.72±1.35	2.72±1.07	0.37±0.28	52.24±3.02
Heart	6.42±3.64	ND	53.38±6.40	2.79±0.78*	0.23±0.48	47.23±4.30*	2.03±1.12	0.42±0.31	23.10±11.61
Liver	5.42±5.06	0.53±0.48*	NDL	2.37±0.37	0.32±0.35	64.02±0.41	2.42±5.16*	0.34±1.02	36.41±9.45
Gizzard	5.26±1.52	NDL	58.24±4.63	5.61±1.23*	0.36±0.27	46.01±1.02	2.04±4.19	0.72±0.56	68.62±11.71*
Blood	ND	0.67±0.56	13.56±10.43	3.54±0.42	0.12±0.25*	4.56±06.64	NDL	ND	19.36±16.46

Values are mean±S.D; *, Values significant at 0.05 level; ND, Not done; NDL, Not detectable level

**Table 2:** Concentration of Heavy Metals in the Internal Tissues and Organs of Poultry Chicken from Present Study and Previous Study

Various organs of Chickens	Similar studies			Range for this study		
	Level of Metals (mg/kg)					
	Pb	Cd	Zn	Pb	Cd	Zn
Intestine	NDL	NDL	NDL	2.49-3.23	0.26-0.42	49.63-83.23
Heart	0.11-0.15^e^	N.De	28.68-36.84^e^	2.9-6.24	N.D-0.27	25.20-46.43
Liver	0.10-0.18^e^	0.007-0.013^e^	18.25-23.21^e^	2.30-5.40	N.D-0.41	64.20-132.21
Gizzard	0.10-0.18^e^	N.De	31.22-48.50^e^	2.10-5.42	N.D-0.54	47.20-75.72
Blood	NDL	NDL	NDL	N.D-2.56	N.D-0.21	4.56-14.36

N.D, Not done; ^e^(Al Bratty et al., 2018); NDL, Not detectable level

## Discussion

Presence of lead in living system has increase the serious health implications such as acute encephalopathy in younger people especially for children below 13 years of age. Prockop et al, (2005) Present analysis depicted high amount of lead content was noted in the blood samples of chicken in selected demographical area. According to Kalogeropoulos et al., (2012) reports depicted that a positive correlation with urbanisation and heavy metal pollution. Lead concentration in liver being higher than in gizzard as previously observed by Bautista, (Boutista et al., 2014).

Moreover, significant difference was noted in the concentration of lead level in the blood as well as various types of organs from the three different districts, this difference due to the various rearing practice in the poultry farm. If the chicken is free range, which may expose it to scavenging waste sites with varying compositions, as well as exposure to polluted sources such as feed, water, containers (Mahaffey, 1977), and sanitation (Bushnell and Jaeger, 1986). As seen in the results, the concentration of lead in the blood of Erode districts, chickens and all of the organs of chickens from the three towns exceeded the overall tolerance limits of 0.1 mg/kg set by the various control organization such as, Egyptian Organisation for Standardization and Quality Control (EOS) (2010), the European Union (2001), the Food and Agriculture Organization, and the World Health Organization, (2000) and 1.0 mg/kg set by the Australia New Zealand Food Authority (2001).

Further, cadmium was the metal found in the least amount in the blood and organs of chickens from the three cities. It was not found in the livers of Erode districts, nor in the hearts or blood of Namakkal area, although it was only found in the intestines of Coimbatore. There is a scarcity of evidence on cadmium in poultry intestines, hearts, and semen with significant other authors. Present analysis shows that, spectrum of values for this sample for intestine and blood, all of which were previously unknown. Related studies’ findings are beyond the ranges obtained in this analysis. Cadmium bioaccumulation in the gizzard has been documented by Nasef and Hamouda (Nasaf and Hamouda, 2008) and Khalafalla et al. (2011). Cadmium has a negative impact on children’s wellbeing (Jarup and Akesson, 2009) and disrupts calcium, phosphate, and bone metabolism (Mamtani et al, 2011). Furthermore, the concentration of cadmium in sections where it was detected exceeded the EOS and EU tolerance limits (0.05 mg/kg) as well as WHO/0.06-0.07 FAO’s mg/kg (29).

Zinc is an important trace factor for protein synthesis (ANZFA, 2001). Poultry meat has been shown to be a source of zinc, which is used as a cofactor in metalloenzymes and to control gene expression (Korish and Attia, 2021). It is needed at a certain amount (35-45 mg/kg) in poultry birds (ANZFA, 2001) for human consumption, as a deficiency causes growth retardation and compromised immune response, among other things. (Korish and Attia, 2021). 

The present study shows that zinc concentrations in the organs and blood of chickens from the three districts is consistent with the statement proposed by Korish and Attia (2020). Sukanya et al., (2016)’s study coincides with the highest amount of zinc contained in the liver of chickens from the three cities. Zinc was also observed in the blood of chickens from Coimbatore and Namakkal, unlike lead and cadmium. Moreover, zinc was significantly different in the same sample type from the three towns. The mean zinc concentration in chicken liver from Coimbatore is higher than the WHO (2000) tolerance limits respectively. Above this threshold, zinc becomes toxic, resulting in copper deficiency, an elevated risk of prostate cancer, and respiratory problems (Bratty et al., 2018; Oyebanji, et al., 2021). 

The findings show that lead, cadmium, and zinc concentrations in organs and blood of local chicken sold at central markets in Coimbatore, Erode, and Namakkal differed in concentration and pattern. Zinc was the only metal found in all five sample types from all three towns; its ratios in each sample form from the three towns were slightly different, and it was the only metal found in all five sample types from all three towns and it was generally the metal with the highest concentration. The order of zinc concentration in organs and blood was liver>intestine>gizzard>heart>blood, with the liver and gizzard having the highest levels of the three metals. Both of the metal concentrations are above the tolerance limits set by certain regular organisations indicating that the chicken samples may pose a health risk to customers.

## Author Contribution Statement

MP contributed to design, methodology, manuscript writing and critical review AM, BB, HKB and ME contributed to data collection and manuscript writing VAA, RS, KD and TP contributed to design and critical review of the manuscript.

## Conflict of interest

None.
